# Taste and odor preferences following Roux-en-Y surgery in humans

**DOI:** 10.1371/journal.pone.0199508

**Published:** 2018-07-05

**Authors:** Hannah Kittrell, William Graber, Evelyn Mariani, Krzysztof Czaja, Andras Hajnal, Patricia M. Di Lorenzo

**Affiliations:** 1 Department of Psychology, Binghamton University, Binghamton, New York, United States of America; 2 Metabolic Surgery, St. Joseph Hospital Health Center, Syracuse, New York, United States of America; 3 Veterinary Biosciences and Diagnostic Imaging, University of Georgia, Athens, Georgia, United States of America; 4 Department of Neural and Behavioral Sciences, Penn State University, College of Medicine, Hershey, Pennsylvania, United States of America; Western University of Health Sciences, UNITED STATES

## Abstract

It is well established that bariatric surgery, the most effective method to achieve long-term weight loss in obese subjects, reverses enhanced preference and intake of sweet/fatty foods. Although taste and odor preference changes following bariatric surgery have been previously described, their time course and relationship to weight loss remains an issue. The aim of this study was to determine the relationship between taste and odor preference changes and successful weight loss following bariatric surgery. A cross-sectional study was performed on 195 human subjects with body mass index (BMI) above 30 (at least class I obesity), who were scheduled to receive (n = 54) or had previously received (n = 141) Roux-en-Y gastric bypass (RYGB). A Self-Assessment Manikin test was used to measure each participant’s affective reaction (ranging from pleasure to displeasure) to a variety of food-related and odor-related pictures. Results confirmed earlier reports about changes in sweet/fatty foods preference after surgery and revealed a shift in preference toward less calorie-dense foods. Relatedly, endorsements of “favorite” foods were mostly sweet/fatty foods in subjects awaiting surgery but were shifted toward more healthy choices, particularly vegetables, in subjects post-RYGB surgery. However, food preference ratings trended toward pre-surgical levels as the time since surgery increased. Answers to open-ended questions about why their diet changed post-surgery revealed that changes in cravings, rather than changes in taste per se, were the major factor. Surprisingly, patients rating a coffee taste as more pleasing after surgery had a lower post-surgical BMI. No associations of odors with change in BMI were apparent. Results showed that following bariatric surgery taste preferences are significantly altered and that these changes correlate with lowered BMI. However, these changes fade as time since surgery lengthens. These results may suggest diagnostic criteria to identify people at risk for less than optimal changes in BMI following bariatric surgery.

## Introduction

The ever-increasing prevalence of obesity is a deadly trend worldwide; it stands neck-and-neck with heart disease and smoking for the leading causes of death. Although dieting by calorie restriction can result in weight reduction, it is notoriously impermanent. In contrast, bariatric surgery has been shown to produce significant and long-lasting weight loss [1, 2] and is now considered the most effective method for the treatment of morbid obesity. Of the several types of bariatric surgery available, Roux-en-Y gastric bypass (RYGB) is one of the most common (http://www.win.niddk.nih.gov/publications/gastric.htm). In RYGB surgery, the size of the stomach is drastically reduced, and the remaining, larger portion of the stomach is attached to the small intestine to drain bile and enzymes. The mechanism(s) that contribute to this weight loss are many and the study of their identification and interactions is at the forefront of research in this area.

It has been suggested that changes in taste preference and/or perception may contribute to altered intake and weight loss after RYGB surgery [3–7]. A recent study, however found no change in taste sensitivity in patients who received RYGB despite altered food preferences [8]. Indeed, multiple studies have shown that following RYGB, patients show changes in food preference away from a high-fat and/or high-sugar diet and evidence a lower recognition threshold for sucrose compared with control subjects [2, 9–12]. Reduced preference and subsequent gradually developing avoidance of foods high in sugar/fat would presumably shift eating habits toward a healthier diet pattern [13–15]. Additionally, it is known that odor plays a role in the sensory and hedonic evaluation of food. However, there is conflicting evidence about whether obesity alters the detection and perception of olfactory stimuli and whether RYGB surgery can affect olfactory perception [16–18].

**Fig 1:**
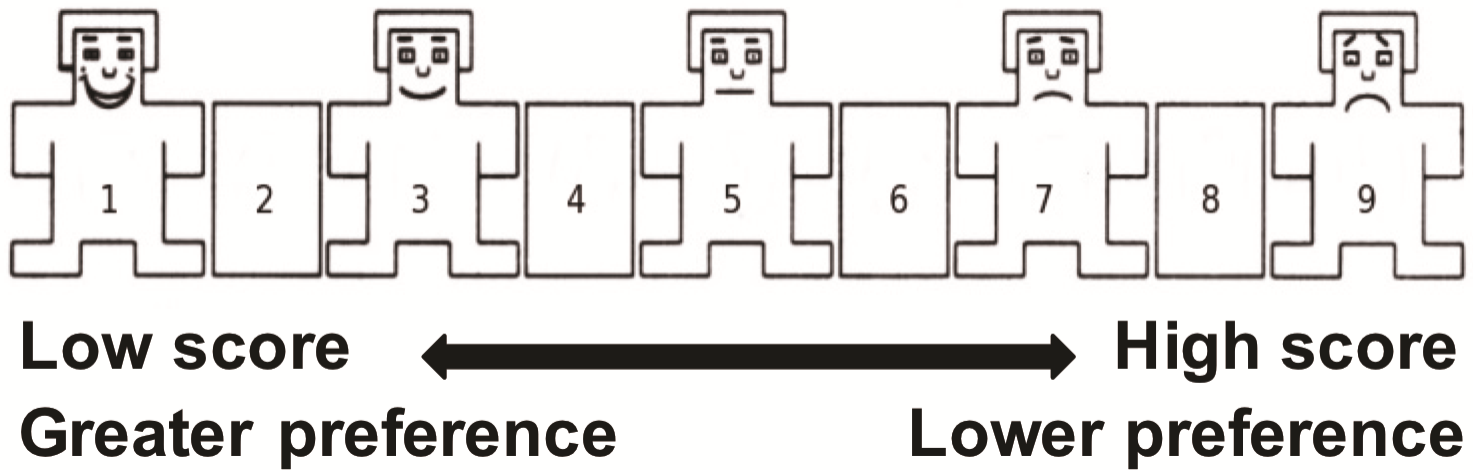
SAM rating guide.

Here we aimed to assess changes in food and odor preference following RYGB surgery and relate them to changes in body-mass index (BMI) and time since surgery. Preference ratings were collected using the Self-Assessment Manikin (SAM) [19] while patients were in the waiting room of a bariatric surgeon’s office. The SAM is a culture free, non-text based, picture-oriented instrument that directly assesses pleasure, arousal and/or dominance associated in response to a specific object. Fig 1 depicts the version of SAM used in this study. Since some patients had not yet undergone surgery, comparisons could be made between those obese patients awaiting RYGB surgery and those who were post-surgery. In our cross-sectional study, we asked patients to rate their preference for a variety of foods with dominant tastes representing the five basic taste qualities (sweet, sour, salty, bitter and umami). In addition, patients rated preference for four odors, two food-related and two non-food-related.

Our findings indicate that RYGB surgery results in lowered preference for sweet and fatty foods compared with obese subjects awaiting surgery, as is well known, but that preference ratings drifted toward pre-surgical patterns by 12 months following surgery. Endorsements of “favorite foods” also changed toward more healthy choices after RYGB surgery, but this effect was lessened as time since surgery increased. Finally, those RYGB patients who rated the odor of coffee as more pleasing after surgery had a lower post-surgical BMI, especially within a year post-surgery. In all, the present study provides support for the interrelatedness of food preferences, changes in diet and changes in BMI following RYGB surgery.

## Materials and methods

The protocol for this study was approved by the Human Subjects Research Review Committee of Binghamton University. Voluntary informed consent was obtained from each subject before entry into the study This included permission to access medical information such as BMI before and after RYGB surgery, time since surgery, smoking status, pregnancy and use of mood altering drugs. (See S1 File).

### Participants

One hundred and ninety-five subjects participated in the study. Subjects were eligible for inclusion in the study if, at one point prior to surgery, their body mass index (BMI, calculated as weight in kilograms divided by height in meters squared) was above 30 (at least class I obesity), and they were scheduled to receive (n = 54) or had previously received RYGB surgery (n = 141). Data collected included BMI before and/or after surgery, age, gender, ethnicity and the date of surgery. Age, gender, ethnicity and the style of bariatric surgery performed, large “open” incision or laparoscopic, were not determining factors in participant selection. Exclusion criteria included the presence of postoperative complications (anemia, infection, dumping syndrome, etc.), pregnancy, presence of alcoholism or smoking.

### Materials

Participants in the study were asked to fill out a questionnaire that assessed taste and olfaction likes and dislikes (see S2 File). A receptionist gave the patients the informed consent form, authorization for release of medical record information, and questionnaires, along with their usual appointment paperwork. The Self-Assessment Manikin (SAM) was used to measure the pleasure associated with the participant’s affective reaction to a variety of food-related and odorant-related pictures [19].

Study participants were asked to provide SAM ratings of 15 food-related and four odor-related pictures. Each of the 15 food-related pictures showed foods that were representative of one of the five basic taste qualities, including sweet, salty, bitter, sour and umami. Two pictures were selected from the International Affective Picture System, and the remaining 13 were selected from a basic Google search of public domain images. These included pictures of a sugar cookie, ice cream, strawberries (all sweet), steak, cheese, chicken (all umami), French fries, potato chips, a soft pretzel (all salty), a lemon, vinegar, sauerkraut (all sour), a cup of coffee, a bar of dark chocolate and a glass of dark beer (all bitter). The same SAM rating scale was used to measure olfactory preferences to 4 odorant-related pictures. Similar to the food-related pictures, the odorant pictures were selected from a basic Google search of public domain images. These included pictures of a cup of coffee, a rose, a banana and a gasoline pump.

Three open-ended questions were included at the beginning of the questionnaire as well. These were: 1) “Have you noticed that your food preferences have changed since surgery? If so, how?”, 2) “What was your favorite food before surgery or, if you haven’t had surgery yet, what is your favorite food now?” and 3) “What is your favorite food now that you have had the surgery?” The first and third questions were skipped if patients were attending a pre-surgical visit and had not yet had surgery.

### Study design

For two one-month periods spaced several months apart, all patients coming into Dr. Graber’s office for a routine pre-surgical or post-surgical office, nutrition or psychology visit were given the option to participate in the study. If a patient wished to participate, the informed consent form and authorization for release of medical record information were completed at the same pre-surgical or post-surgical visit, followed by completion of the questionnaires on food and olfaction preference. Completion of both questionnaires took no longer than 10 minutes. Patients who were attending a pre-surgical visit only qualified for the study if they subsequently underwent bariatric surgery at a later date. Thus, this was a cross-sectional study with no follow up once all questionnaires were completed.

Participants filled out the questionnaires individually within Dr. Graber’s practice. Subjects were instructed that a series of food-related pictures would be presented to them, and that he or she would be rating each picture in terms of how it made them feel while viewing it. Each subject was asked to imagine how he or she would feel while eating the food presented in the picture, and make the rating based on their immediate personal experience and no more. Similar instructions were used for the olfaction preference questionnaire, with the exception that subjects were asked to imagine how he or she would feel while smelling the odorant implied by the picture.

### Statistical analysis

A power analysis was performed using R to determine the appropriate sample size needed for this study. A good estimate for what the effect size might be could not be found in previous literature, so the power analysis was calculated across a wide range of effect sizes and the result was plotted as a curve of sample size (per group) vs. effect size. Estimating a medium effect size of 0.3, it was determined that about 25 subjects per group would be adequate, for a total of 125 subjects in the study, with a power of 0.8 at the 0.05 significance level.

An unpaired *t* test was performed to determine the difference between SAM ratings pre- and post-operatively. Spearman’s *rho* correlation coefficients were calculated to determine the relationship between food and odorant preferences and time since surgery, pre-surgical BMI, post-surgical BMI and change in BMI. Analysis was performed using XLSTAT 2016.1 software for Microsoft Excel. Significance was corrected for multiple comparisons using the False Discovery Rate method [20].

## Results

### Subject demographics

Of the 195 subjects who participated in this study, 54 were awaiting RYGB surgery and 141 were post RYGB surgery. Among those awaiting surgery, there were 47 female (mean age = 46.3 ± 1.5 yrs; median age = 49.5 yrs) and 7 male (mean age = 55.6 ± 1.9 yrs; median age = 56 yrs) subjects. Male subjects were significantly older than female subjects (Student’s *t* test, *p* < 0.03). For those subjects who had undergone RYGB surgery, there were 108 female (mean age = 45.7 ± 1.1 yrs; median age = 45 yrs), 20 male (mean age = 51.7 ± 2.7 yrs; median age = 52.5 yrs) and 13 subjects who declined to submit demographic data. Both subject groups were overwhelmingly white: among those awaiting surgery and who indicated their race/ethnic origin, 50 subjects were white, 2 African-American, 1 Hispanic and 1 unknown; among those who had undergone RYGB surgery and who indicated their race/ethnic origin, 119 (97%) were white and 4 African-American. The proportion of subjects who were taking mood-altering drugs was lower in subjects who had undergone RYGB surgery (53/141, 38%) compared with subjects who were awaiting RYGB surgery (26/54, 48%). This difference is not significant (chi-square = 1.91, *p* = 0.17)

### RYGB surgery—Effects on BMI

Average BMI did not differ (Student’s *t* test, *p* = 0.8) between subjects who have not yet had RYGB surgery (mean BMI = 47.9 ±1.1; median BMI = 47.4) and the BMI of subjects before having RYGB surgery (mean BMI = 48.3 ± 0.9; median BMI = 46.7). Following RYGB surgery, at the time the survey was taken (mean time since surgery = 35.7 ± 3.1 SEM months; median = 24 months), BMIs averaged 35.7 ± 0.9 (median = 34.3). This difference was significant (Student’s *t* test, *p* < 0.01).

**Fig 2:**
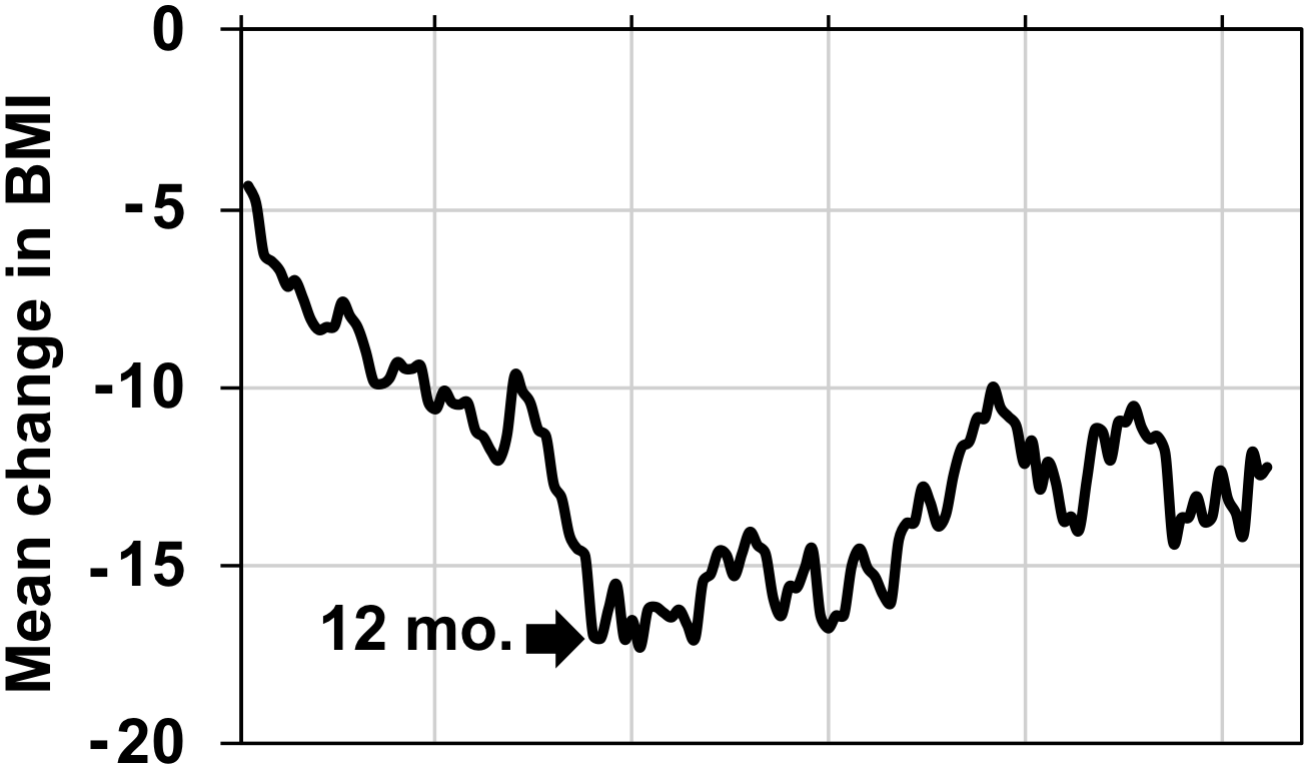
Relationship between time since RYGB surgery and change (reduction) in BMI. Results of a moving average of change in BMI across the sample. Each point was the average of 10 subjects; the average was then advanced by one subject and recalculated. The point at which the slope of this curve changed was at 12 months post-surgery.

Overall, there was a 0.3 correlation (*p* > 0.05) between the time since surgery and change (reduction) in BMI pre-post surgery. To track the degree of weight loss with respect to time since surgery, we sorted subjects according to the time since surgery and calculated a moving average of reductions in BMI (n = 10 subjects for each average, advanced by one subject and recalculated). Fig 2 shows the results of this analysis. It is apparent that the slope of the curve, representing the rate of reduction in BMI, changes at approximately 12 months post-surgery. We therefore divided the sample into those subjects who had undergone RYGB surgery in the last 12 months (*n* = 53; 37 females; 10 males; 6 declined to specify) and those who had had RYGB surgery more than 12 months ago (*n* = 88; 68 females; 11 males; 9 declined to specify). (We discarded data from three female subjects who had undergone RYGB surgery less than a month ago.) There was a significant positive correlation (*r* = 0.79, *p* < 0.001) between the time since RYGB surgery and the reduction in BMI only for those subjects who had undergone surgery within the past 12 months. For those subjects who had undergone RYGB surgery more than 12 months ago, this correlation, which was not statistically reliable, was 0.14 (*p* > 0.05), In all, results show that RYGB surgery results in a reduction in BMI which generally stabilizes the longer the time since surgery.

### RYGB surgery—Effects on food and odor preference in open-ended questions

Eighty-eight subjects (88/141, 62%) responded to the open-ended questions about changes in food preferences and/or favorite foods following RYGB surgery. Seventy-two (72/88, 82%) said that their food preferences and/or favorite foods have changed since the surgery; sixteen (16/88, 18%) did not notice a change. Whether food preferences changed after surgery was not statistically associated with BMI prior to surgery, present BMI or the amount of reduction in BMI since RYGB surgery. However, those postsurgical subjects who indicated that their food preferences changed had undergone surgery significantly more recently (mean time since surgery = 38.4 ± 4.6 months) than subjects who said that their food preferences did not change (mean time since surgery = 59.4 ± 15.5 months) (Wilcoxon signed rank test, *p*<0.05). Although few of the subjects *explicitly* commented on the effects of time on their food preferences, the three who did are worth noting. Two subjects reported changes in food preference immediately following surgery, but noted that after a while, their preferences returned to normal: “Sweet foods seem sweeter and I’ve had foods such as pulled pork that I loved before I didn’t like after surgery. But 2 years after surgery everything has gone back to the way it was pre-surgery”, and “Yes, [I noticed food preference changes] very much for the first 8 months, portions and most any red meat made me sick. Not now though, one year out I can eat anything.” In contrast to these remarks, the final patient had quite the opposite experience, noting that “Yes, my food preferences have changed very much so, and this is still after having surgery 10 years ago.” In general, however, these results suggest that changes in food preferences following RYGB surgery may happen, but in most cases fade with time.

**Fig 3:**
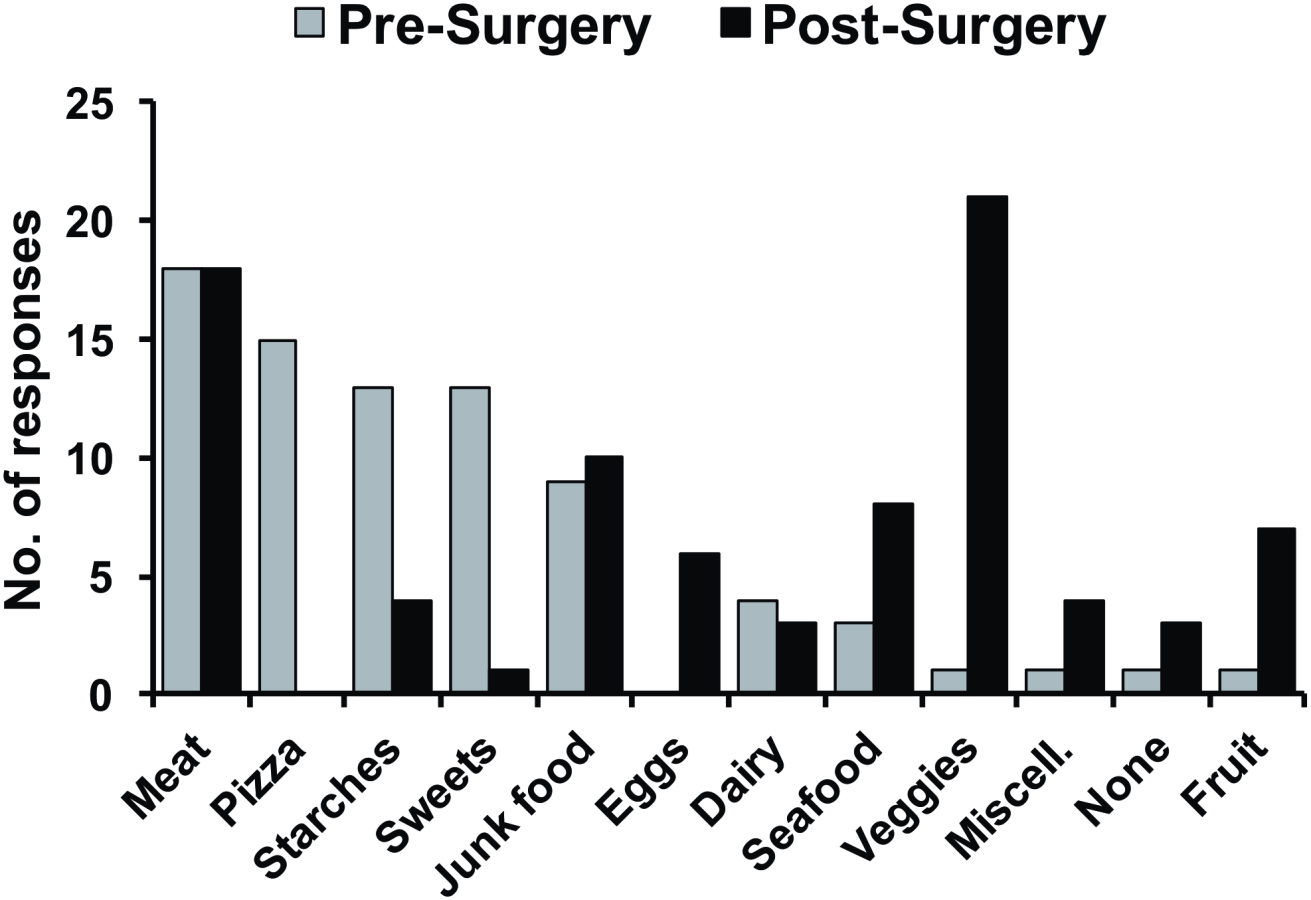
Responses to open ended question about favorite foods prior to and after surgery.

In lieu of tracking food intake in our subjects, we asked for their “favorite” foods pre and post-surgery based on the assumption that favorite foods would reflect food preference. Fig 3 shows the types of favorite foods before and after surgery for those subjects who responded. In general, pizza, starches and sweets were endorsed as the favorite food by more subjects prior to surgery than after, but seafood, vegetables and fruit were favored by a greater proportion of subjects after surgery than prior. Pre-surgically, the indication of “meat” as a favorite food referred specifically to red meat that tends to be less healthful such as steak, prime ribs, bacon or hamburgers. Of the 22 subjects who reported meat as a favorite food pre-surgically, 7 (32%) were referring to poultry, while 15 (68%) were referring to red meat. After surgery, however, favorite meat was mostly grilled or baked chicken. Of the 27 subjects who reported meat as a favorite food post-RYGB, 17 (63%) were referring to poultry, while 10 (37%) were referring to red meat.

Nutritional management is an important factor in sustaining long-term weight loss for post-surgical bariatric patients. Protein malnutrition is often an issue after bariatric surgery, as many foods high in protein are also energy-dense and high in fat, and cause “dumping” symptoms including abdominal pain and cramping, nausea, and diarrhea. As such, it is recommended that post-surgical patients consume lean protein sources, including chicken, fish, eggs, lean beef or pork, and low-fat dairy. The shift of favorite meat foods post-RYGB toward poultry and away from red meats is in line with this dietary advice. As it is important to get adequate nutrition post-surgically without overeating, avoiding sweets and junk foods, which are high in calories but offer little nutritional value, is also important. Again, subjects in this study seemed to be following these guidelines. Pre-surgically, 24 subjects (24/72, 33%) indicated junk food (pizza, French fries, Chinese food, macaroni and cheese) and 18 subjects (18/72, 25%) indicated sweets (ice cream, cake, cookies, chocolate) as favorite foods. Post-surgically, only 7 subjects (7/72, 10%) and 1 subject (1/72, 1%) indicated junk foods and sweets as favorite foods, respectively. In contrast, vegetables and fruits were more likely to be designated as a favorite foods post-surgery. Only 2 subjects (2/72, 3%) indicated fruits and vegetables as a favorite food pre-surgery, while 26 subjects (26/72, 36%) and 13 subjects (13/72, 18%) indicated vegetables and fruits, respectively, as favorite foods post-surgery.

Table 1 shows the proportion of subjects who indicated how/why their food preferences changed after surgery (n = 71) for each of several reasons. (These reasons were grouped into broad categories by two of the experimenters (HK and PMD) independently and condensed as Table 1.) Categorizations were made based on key words in the comments, as follows: “Taste” statements included the specific word taste; “Trying to be Healthy/Listening to Doctor” statements included variations of the word “health,” “doctor,” or “staying on track;” “Foods are Nauseating/Unsettling” statements included words indicative of such malaise, including “puke,” “bloated,” “stomach ache,” etc.; “Change in Craving/Wanting of Food” statements included variations of the words “crave,” “desire” or “want;” and “Like different Foods” statements included variations of the words “like,” “love,” or “enjoy.” For comments that included aspects of more than one categorization, the research team discussed as a group which category was most appropriate. In general, most subjects who volunteered these insights changed the kinds of foods they enjoyed. However, many subjects could no longer tolerate some categories of foods, such as dairy, and many came to dislike greasy (fried) foods (see Table 1).

**Table 1:** Stated causes of subjects’ taste/food preference changes following RYGB surgery.

Cause of Preference Change	Comments	No. of Subjects (%)
Taste	“Tomatoes don’t taste right”, “Most foods taste bad or have no taste”, “Everything tastes salty to me”, “Sweet foods are sweeter”	9/71 (13%)
Trying to be Healthy/Listening to Doctor	“I have a desire to eat healthier”, “I am afraid of hurting myself so I stick to the list from my doctor”, “I eat completely different now only because it is important to stay on track, I don’t take my surgery lightly”, “Making healthy choices is how I eat now”	8/71 (11%)
Foods are Nauseating/Unsettling	“Some foods like bread make me puke, “I don’t gravitate to starch items as much because it makes me feel bloated”, “Food goes through me faster”	14/71 (20%)
Change in Craving/Wanting of Food	“I no longer crave sweets”, “I no longer crave fast food”, “I find that I crave fruits more now”, “I can say no!”, “I was a soda and ice cream nut, I can’t eat either anymore because I have no desire”	13/71(18%)
Like different Foods	“I used to love meat now I force myself to eat it”, “I enjoy some sweets less”, “I now like spicy, ‘hot’ foods”	27/71 (38%)

Finally, there were also three patients who commented on the effect of bariatric surgery on their smell, and how this affected their food preferences, even though they were not prompted to do so. These comments were: “Foods I used to enjoy now the smell turns me off,” “I can’t stand the smell of any fast food now,” and “Certain smells are upsetting to me now—fish, bathroom soap and perfume/cologne smell too strong.” Common to all three statements is the idea that certain smells increased in intensity, so much so that they actually became aversive. Additionally, food items associated with these aversive smells were no longer tolerated. This suggests strong odors and odor preferences interact with specific food preferences and can lead to food aversions. Conversely, changes in food preferences can produce changes in odor preference.

### RYGB surgery—Effect on preference ratings of food and odor

**Fig 4:**
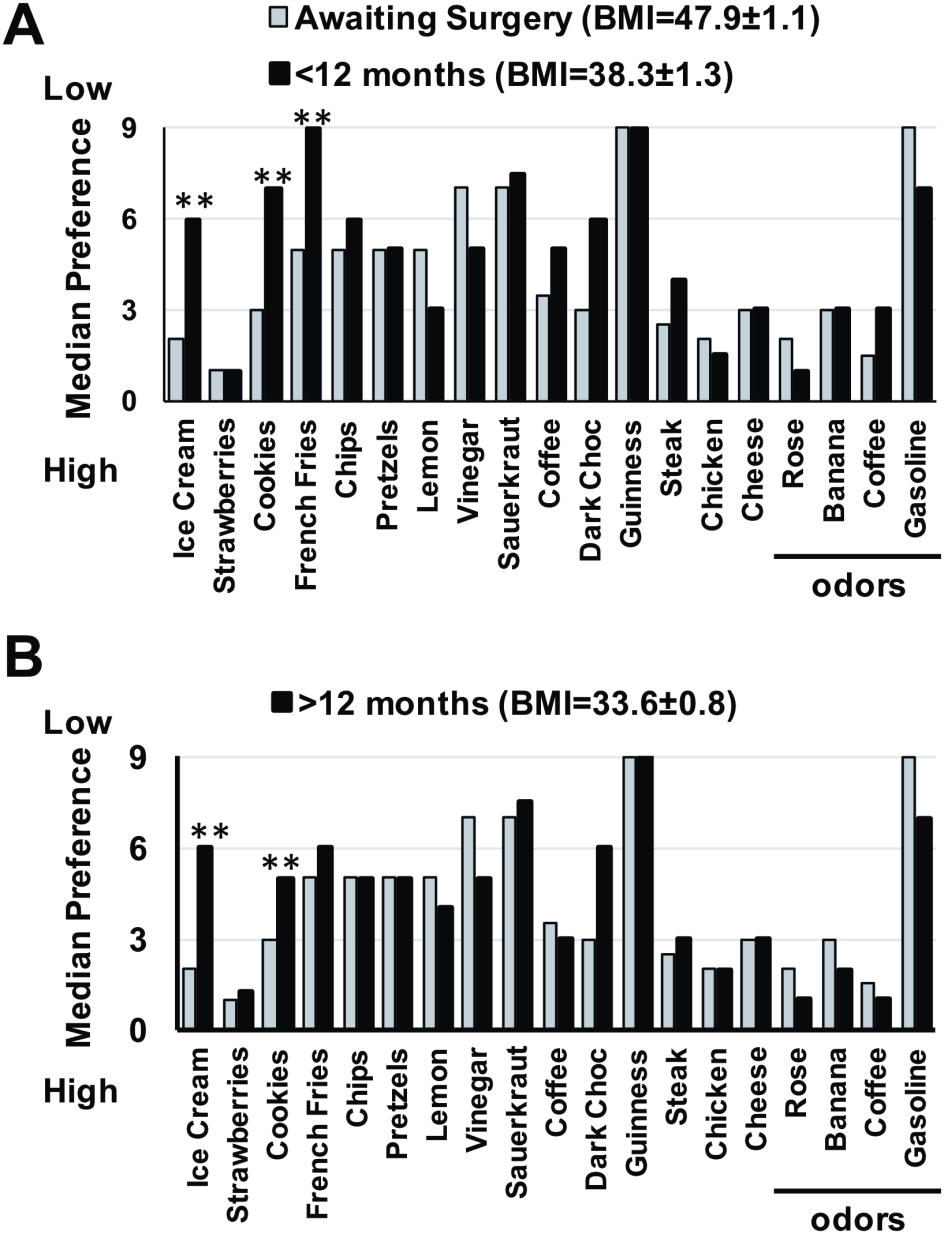
Median preference ratings for all pictures representing taste and odor stimuli for those awaiting RYGB surgery and those who have had RYGB surgery. A. Subjects who had RYGB surgery less than 12 months prior to the survey. B. Subjects who had RYGB surgery more than 12 months prior to the survey. **p < 0.01.

Fig 4 shows the median preference ratings for taste and odor stimuli in subjects awaiting surgery compared with those who had surgery less than 12 months ago (Fig 4A) and those who had surgery over 12 months ago (Fig 4B). Wilcoxon Signed Rank tests were used to compare the median preference ratings; an alpha level of 0.002 was used to compensate for multiple comparisons (Bonferroni correction). Results showed that, for those subjects who had undergone RYGB surgery within 12 months, preference ratings for ice cream, cookies and French fries were lower (higher numbers) than those of subjects awaiting surgery; for those subjects who had had surgery over 12 months ago, preference ratings for ice cream and cookies, but not French fries were lower (higher numbers). There were significant differences in the preference ratings for cookies and French fries between the group with more recent surgery and those who had undergone surgery over a year ago (Fig 4B), suggesting that RYGB subjects’ preference for these sweet and high fat foods drifted closer to those of subjects awaiting surgery. Consistent with these results are data showing that the longer the time since surgery, the better the preference ratings for cookies (*rho* = -0.26, *p*< 0.002) and French fries (*rho* = -0.29, *p* < 0.001).

Following RYGB surgery those subjects whose BMI changed the most showed a greater preference specifically for the taste (but not the smell) of coffee (*rho* = -0.25, *p* < 0.005). Moreover, at the time of the survey, those subjects with lower BMIs had the most favorable preference ratings for coffee (*rho* = 0.24, *p* < 0.006). When only those subjects who had undergone RYGB surgery within a year prior to the survey were considered, the correlation between the amount of reduction in BMI and the enhanced preference for coffee taste was even more prominent (Reduction in BMI vs. SAM rating for coffee taste: *rho* = -0.45, *p* < 0.002) and it increased the longer the time since surgery (Time since surgery vs. SAM rating for coffee taste: *rho* = -0.57, *p* < 0.0001). Conversely, in those subjects who had undergone surgery over a year ago, the relationship between preference ratings for any taste or odor stimulus and BMI before or after RYGB surgery, or the reduction in BMI, was no longer apparent. There were no significant correlations between BMI and any taste or odor SAM ratings among those subjects awaiting surgery.

## Discussion

Results of a survey of 195 subjects (141 who had undergone RYGB surgery, 54 awaiting surgery) were consistent with the literature showing that RYGB affects taste preferences [15, 21–23], but shed new light on the specificity and time course of these changes. The majority (62%) of postsurgical subjects reported that their food preferences had changed after RYGB surgery. Whether or not subjects reported a change in food preferences was not related to changes in BMI but rather correlated with the time since surgery; those who had had surgery more recently were significantly more likely to report changes in food preferences. Results also showed that likability of specific foods and food categories were modified following RYGB surgery. Specifically, “favorite foods” before surgery were sweet, starchy and fatty foods but in many cases switched to more healthy choices (e.g. vegetables) after surgery, consistent with previous findings [24]. The stated reasons for this change varied, but only 20% of subjects indicated a change in tolerance for some categories of food such as meat and dairy. SAM ratings for specific foods such as ice cream and cookies were significantly less favorable after RYGB surgery; ratings for French fries were poorer only in the group that had undergone RYGB surgery within the year prior to the survey. Interestingly, there was a positive significant correlation between preference for the taste of coffee (but not any other food or odor) and change in BMI in the year following RYGB surgery. This association was no longer apparent in subjects who had undergone RYGB surgery more than a year prior to the survey. In all, present results show that food preferences change following RYGB surgery but these changes generally fade, or at least become less pronounced with time.

It has been well established that there is a period of rapid weight loss immediately following RYGB surgery that lasts for about a year followed by a period of weight stability or weight gain [25]. Our data confirm this observation. Moreover, we also found other sequelae of RYGB surgery that were most pronounced in the first postsurgical year. For example, reports of changes in taste preference were more common during the first year vs. later periods following RYGB surgery. However, the possibility exists that it is the *awareness* of changes in food preference that fades with time since surgery, and not taste preference *per se*. Related to this issue, taste preference ratings for some foods, particularly highly caloric and palatable foods, were diminished in the first postsurgical year but trended toward a return to presurgical levels in subjects whose surgery was over a year ago. These results suggest that changes in taste preference do fade with time since surgery, consistent with the literature [4]. Furthermore, the findings support a recent clinical report that taste changes may not be the sole contributor to altered food preferences, particularly after an extended period following RYGB surgery [8].

Although taste preference changes have been shown to occur following bariatric surgery, the time course and mechanisms behind these changes are still unknown. Burge et al. [11] assessed taste thresholds for sucrose and urea as well as taste preferences before RYGB surgery and at 6 and 12 wks post-surgery. Results showed that sucrose (but not urea) thresholds increased post-surgery. A post-surgical aversion to meat has also been reported [11, 21]. In the present study, only 20% of subjects (14 of 71subjects responding) reported aversions to some foods after RYGB surgery. In a study by Graham et al. [21], 73% (75 of 103 subjects) reported a change in taste perception after RYGB surgery, with approximately equal percentages of subjects reporting either an increase or decrease of perceived intensity for sweet, salty or bitter foods. In contrast, only 13% (9 of 71 subjects) noted a change in taste in the present study when asked in open-ended questions about how their food preferences changed after surgery. However, in addition, 18% (13 of 71) of postsurgical RYGB subjects reported a change in cravings/wanting and 38% (27 of 71) noted a change in liking of different foods in our study. These results are consistent with previous findings [2, 8, 12] suggesting that RYGB surgery decreases the reward value of palatable food. Collectively, these data suggest that a change in taste alone is not the major factor determining alterations of food choice following RYGB surgery.

The association with preference for the taste of coffee and change in BMI presents a puzzle. Consumption of caffeinated beverages is typically discouraged following RYGB surgery, especially in the early months. Importantly, our results relate only the relationship of the *stated preference rating* for coffee, not coffee consumption, to successful weight loss. One possible explanation is that the deprivation of coffee in habitual coffee drinkers induces a craving, thus accounting for an increase in preference rating [26]. Thus, those who were more compliant in restricting coffee consumption might have also been compliant in other postsurgical dietary recommendations, leading to more successful weight loss. Even if that were true, it remains unclear as to why postsurgical deprivation of much more palatable foods, such as ice cream or French fries, would not also lead to cravings and increased preference ratings. Just the opposite result was found. That is, RYGB surgery decreased preferences for these foods but without an association with weight loss. Another potential explanation for the association between preference for coffee and successful weight loss is that the tolerance for bitter tastes is enhanced. Consistent with this notion is the observation to preference for vegetables, which can be slightly bitter, increases in many RYGB subjects following surgery.

It is known that odor plays a role in sensory and hedonic evaluation of food, and that obesity alters the detection and perception of olfactory stimuli [16–18]. However, the exact mechanisms of how obesity modulates olfaction remain unresolved. Few studies have investigated the effects of bariatric surgery on olfaction preference and the possible role this plays in taste preference change. There are conflicting reports in the literature about whether one’s sense of smell is affected by RYGB surgery. For example, Graham et al. [21] reported that 42% (43 of 103) of subjects noted changes in smell following RYGB surgery, though exactly how their sense of smell was altered or whether changes in smell correlated with amount of weight loss was not reported. The negative findings in the present study may be a function of some limitations imposed by the brief questionnaire that we used. That is, we only assessed *preference* ratings for four odorants: two that were food-related (banana and coffee) and two that were non-food-related (rose and gasoline). It is possible that, had we tested a more extensive array of odorants, changes in odor preference may have become apparent. We did not ask about the changes in perception of odor intensity. However, as in Graham et al.’s [21] study, some subjects spontaneously commented that foods smelled more intense, and became aversive. These data provide a hint that there may be changes in sensitivity to odor intensity that may then lead to dietary modifications after RYGB surgery.

## Conclusions

Surveys of a large (n = 195) cohort of patients either pre- (n = 54) or post- (n = 141) RYGB surgery showed that changes in taste cravings, rather than taste *per se*, were subjectively attributed to changes in dietary choices post RYGB surgery. Changes in food preferences were significantly correlated with successful weight loss in the first year post- RYGB surgery. Food preference ratings trended toward pre-surgery levels after the first year following RYGB surgery, suggesting that RYGB patients for whom altered taste preferences are impermanent may be most at risk for weight regain.

## Supporting information

S1 File Appendix A. Informed consent. This document was given to all subjects prior to testing for food and odor preference ratings.
(DOCX)

S2 File Appendix B. Questionnaire. Food and odor preference questionnaire with open-ended questions and a SAM rating section.
(DOCX)

S1 Table Appendix C. Original data. All data collected for all subjects.
(XLSX)
