# Supplementary material for: Taste and odor preferences following Roux-en-Y surgery in humans
Source: PLoS One. 2018 Jul 5;13(7):e0199508. doi: 10.1371/journal.pone.0199508 (PMC6033408; doi:10.1371/journal.pone.0199508)
Supplement: S1 File — This document was given to all subjects prior to testing for food and odor preference ratings. (DOCX) [file pone.0199508.s001.docx]

**S1 File. Appendix A. Informed consent.**  This document was given to all subjects prior to testing for food and odor preference ratings.

**INFORMED CONSENT**

Project Director or Principal Investigator ____Hannah Kittrell________________

Title of Project __ Timing of Taste Preference Change following Bariatric Surgery and its Effect on Successful Weight Loss

You are invited to participate in a research study of taste preference changes following bariatric surgery. We hope to learn exactly when these taste preference changes occur post-surgically, and if the timing of the changes has any correlation with weight regain following surgery**.** The only experimental procedures include completing two brief questionnaires. You were selected as a possible participant in this study because you are a qualifying bariatric surgery patient. There are approximately 125 subjects involved in the study.

If you decide to participate, we will ask you to complete a brief (15 item) questionnaire on taste preferences and a brief (4 item) questionnaire on smell at this visit. The purpose of the completion of these questionnaires is to determine individual differences in attitudes toward different types of food. Completion of both questionnaires will take no more than 15 minutes. Discomforts, inconveniences and risks reasonably to be expected include feeling any sort of discomfort from completing a brief questionnaire on dietary/eating habits. We cannot and do not guarantee or promise that you will receive any benefits from this study. However, participation in this study will help to bring more knowledge to the scientific area of bariatric surgery and weight loss. Specifically, studies of the mechanisms by which bariatric surgery changes taste may aid in the development of nonsurgical therapeutic methods that will still promote successful and long-lasting weight loss.

In order to ensure confidentiality and anonymity of subjects, assigned code numbers will replace names, only Dr. Graber and his employees will know which names are associated with which code numbers. Access to data collected will be limited to Dr. Graber and his employees, the principal investigator, Hannah Kittrell and the faculty supervisor, Dr. Patricia Di Lorenzo, and all data will be stored digitally in a password-protected file. If you give us your permission by signing this document, we plan to disclose all information pertaining to the study including answers to both questionnaires, as well as demographic information such as BMI before and/or after surgery, dates of surgery, age, ethnicity, gender, and the presence of some comorbidities including smoking, alcoholism, pregnancy, postoperative complications, depression and severe anxiety. This research will remain confidential unless we are required by New York State Law to report harm to yourself.

You will not receive any sort of fee for participating in this study, and we do not foresee any additional costs that may result from participation in this study.

In the event of a research related injury, please contact:

1. Hannah Kittrell (Principal Investigator): 585-402-9202, [hkittre1@binghamton.edu](mailto:hkittre1@binghamton.edu)
2. Patricia Di Lorenzo (Faculty Supervisor): [diloren@binghamton.edu](mailto:diloren@binghamton.edu)
3. Dr. William Graber: 315-477-4740

Your decision whether or not to participate will not prejudice your future relations with the office of Dr. William Graber and Binghamton University. Your participation is voluntary, if you decide to participate; you are free to withdraw your consent and to discontinue participation at any time without prejudice. There will be no consequences should you decide to withdraw from the research study. If a participant requests that we do not use his/her study information, we will destroy all records. If the participant requests this after beginning some of the data collection, all records of survey answers will be destroyed.

Any significant new findings developed during the course of the research which may relate to the subject’s willingness to continue participation will be provided to the subject.

Before you sign the form, please ask questions on any aspect of the study that is at all unclear to you. If you have any additional questions, concerns, or complaints later or wish to report a research related problem, Hannah Kittrell, 585-402-9202, hkittre1@binghamton.edu, will be happy to answer them. If at any time you have questions concerning your rights as a research subject or you have questions, concerns, or complaints about the research you may call Binghamton University's Human Subject's Research Review Committee at (607) 777-3818. You will be given a copy of this form to keep.

If you would like to receive the results of this study, please contact Patricia Di Lorenzo, [diloren@binghamton.edu](mailto:diloren@binghamton.edu), expressing your interest.

YOU ARE MAKING A DECISION WHETHER OR NOT TO PARTICIPATE. YOUR SIGNATURE INDICATES THAT YOU HAVE DECIDED TO PARTICIPATE HAVING READ THE INFORMATION PROVIDED ABOVE.

Date _____________

Signature __________________________________________
