# Supplementary material for: Taste and odor preferences following Roux-en-Y surgery in humans
Source: PLoS One. 2018 Jul 5;13(7):e0199508. doi: 10.1371/journal.pone.0199508 (PMC6033408; doi:10.1371/journal.pone.0199508)
Supplement: S2 File — Food and odor preference questionnaire with open-ended questions and a SAM rating section. (DOCX) [file pone.0199508.s002.docx]

**S2 File. Appendix B**. Food and odor preference questionnaire with open-ended questions and a SAM rating section.

**Bariatric Surgery and Taste Preference**

We thank you for coming today and appreciate your participation in this experiment. For about the next 10 minutes, you will be looking at different pictures on the papers in front of you, and you will be rating each picture in terms of how it made you feel while viewing it. There are no right or wrong answers, so simply respond as honestly as you can.

Before you start the picture-rating portions of this questionnaire, we ask that you take a few minutes to answer the following questions.

1. Have you noticed that your food preferences have changed since having bariatric surgery? If so, how? *Skip this question if you have not had surgery.
2. What was your favorite food before surgery or, if you haven’t had surgery yet, what is your favorite food now?
3. What is your favorite food now that you have had the surgery? *Skip this question if you have not had surgery.

**Food Preference Questionnaire**

If you will look at the questionnaire below, you will see a set of 5 figures arranged along a continuum from 1 to 9; you will be using these figures to rate how you felt while viewing each picture. If you feel that a particular food item is extremely pleasant or satisfying you can indicate this by placing an “X” over figure 1. You can indicate feeling completely unhappy or unsatisfied by a food item by placing an “X” over figure 9. You can also describe intermediate feelings of pleasure, by placing an “X” over any of the other figures, or boxes in between the figures. Please use the set of Self-Assessment Manikin (SAM) figures BELOW each image to rate the image.

When rating the pictures, we ask that you imagine how you would feel while **eating** the food presented in the picture, and make your rating based on this. Your rating of each picture should reflect your immediate personal experience, and no more. Please rate each picture as you actually felt while you viewed the picture.

Pictures of a sugar cookie, ice cream, strawberries, steak, cheese, chicken, French fries, potato chips, a soft pretzel, a lemon, vinegar, sauerkraut, a cup of coffee, a bar of dark chocolate and a glass of dark beer were inserted here.

**Odorant Preference Questionnaire**

Similar to the previous section, in the questionnaire below you will be looking at different pictures on the paper in front of you, and you will be rating each picture in terms of how pleasant or unpleasant it made you feel while viewing it. The same set of 5 SAM figures will be used to rate the pictures below. Please use the set of SAM figures BELOW each image to rate the image.

When rating the pictures below, we ask that you imagine how you would feel while **smelling** the item presented in the picture, and make your rating based on this. Your rating of each picture should reflect your immediate personal experience, and no more. Please rate each picture as you actually felt while you viewed the picture.

Pictures of a cup of coffee, a rose, a banana and a gasoline pump were inserted here.

FOR OFFICE USE ONLY:

Questionnaire #: ______

Patient Name:
